# Detecting Early-Stage Cohesion Due to Calcium Silicate Hydration with Rheology and Surface Force Apparatus

**DOI:** 10.1021/acs.langmuir.2c02783

**Published:** 2022-11-25

**Authors:** Teresa Liberto, Andreas Nenning, Maurizio Bellotto, Maria Chiara Dalconi, Dominik Dworschak, Lukas Kalchgruber, Agathe Robisson, Markus Valtiner, Joanna Dziadkowiec

**Affiliations:** † Institute of Materials Technology, Building Physics and Construction Ecology, Faculty of Civil Engineering, Vienna University of Technology, 1040 Vienna, Austria; ‡ Institute of Chemical Technologies and Analytics, Vienna Institute of Technology, 1060 Wien, Austria; § Opigeo SRL, 36040 Grisignano di Zocco, Italy; ∥ Department of Geoscience and CIRCe Center, University of Padua, 35131 Padova, Italy; ⊥ Institute of Applied Physics, Vienna Institute of Technology, 1040 Wien, Austria; # NJORD Centre, Department of Physics, University of Oslo, P.O. Box 1048, Oslo 0316, Norway

## Abstract

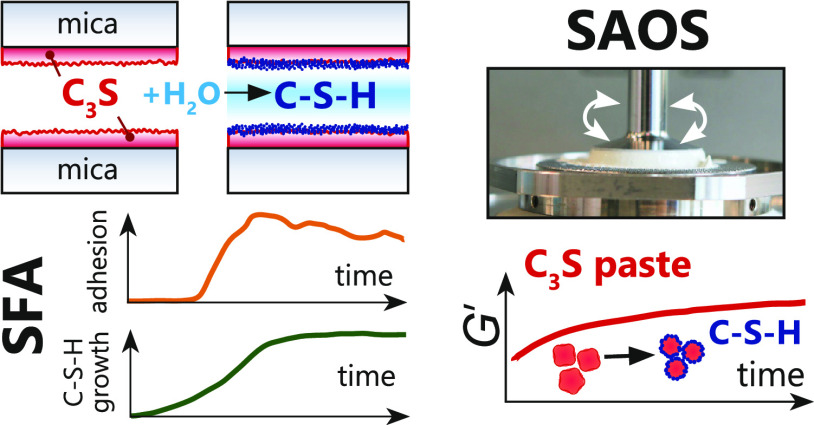

Extremely robust cohesion triggered by calcium silicate hydrate (C–S–H) precipitation during cement hardening makes concrete one of the most commonly used man-made materials. Here, in this proof-of-concept study, we seek an additional nanoscale understanding of early-stage cohesive forces acting between hydrating model tricalcium silicate (C_3_S) surfaces by combining rheological and surface force measurements. We first used time-resolved small oscillatory rheology measurements (SAOSs) to characterize the early-stage evolution of the cohesive properties of a C_3_S paste and a C–S–H gel. SAOS revealed the reactive and viscoelastic nature of C_3_S pastes, in contrast with the nonreactive but still viscoelastic nature of the C–S–H gel, which proves a temporal variation in the cohesion during microstructural physicochemical rearrangements in the C_3_S paste. We further prepared thin films of C_3_S by plasma laser deposition (PLD) and demonstrated that these films are suitable for force measurements in the surface force apparatus (SFA). We measured surface forces acting between two thin C_3_S films exposed to water and subsequent in situ calcium silicate hydrate precipitation. With the SFA and SFA-coupled interferometric measurements, we resolved that C_3_S surface reprecipitation in water was associated with both increasing film thickness and progressively stronger adhesion (pull-off force). The lasting adhesion developing between the growing surfaces depended on the applied load, pull-off rate, and time in contact. These properties indicated the viscoelastic character of the soft, gel-like reprecipitated layer, pointing to the formation of C–S–H. Our findings confirm the strong cohesive properties of hydrated calcium silicate surfaces that, based on our preliminary SFA measurements, are attributed to sharp changes in the surface microstructure. In contact with water, the brittle and rough C_3_S surfaces with little contact area weather into soft, gel-like C–S–H nanoparticles with a much larger surface area available for forming direct contacts between interacting surfaces.

## Introduction

Concrete materials based on Portland cement (constituted mainly by soluble tricalcium, C_3_S, and dicalcium, C_2_S, silicates^1^) are among the most used man-made materials owing to the remarkably robust and unprecedented cohesive properties developing during cement setting and hardening.^2^ Once exposed to water, the constituent calcium silicate grains dissolve and are partially replaced by hydrated mineral phases, together forming a complex composite material. The main hydration products are the gel-like, quasi-amorphous calcium silicate hydrates C–S–H^a^ and the highly crystalline calcium hydroxide CH.^1^ C–S–H starts to precipitate within a few minutes from contact with water, while CH starts to precipitate after a few hours, when the calcium saturation level in the pore solution is reached.^3,4^ In later stages of hydration, the C–S–H nanoparticulate gel spreads into the open pore spaces, forming a progressively densifying, platy, 3D microstructure.^1,5−10^ During hydration reactions and carbonation, the pH of the interstitial solution decreases from about pH ≈ 13 to a final value that may attain pH ≈ 9. This is a slow diffusive process (i.e., years), which proceeds via successive layers of degradation, where pH buffering is exerted successively by Portlandite (CH), C–S–H, layered double hydroxides, ettringite, and calcite.^11,12^

This partial dissolution of water-exposed cement grains and formation of C–S–H nanohydrate gel govern cement cohesion.^9,13−17^ In the early stage of hydration, the cohesiveness of cement paste is regulated by the ionic composition of the interstitial solution.^14,18,19^ There, the high number of ions (such as Ca^2+^ ∼20 mM, OH^–^, H_2_SiO_4_^2–^)^20,21^ and the high C–S–H surface charge caused by silanol surface groups’ deprotonation at high pH lead to the development of strongly attractive interactions acting at short separation distances of a few nanometers.^14,19^ The pronounced dependence of interparticle interactions within cement and thus cement coagulation on dissolved Ca^2+^ content have been verified with electrokinetic studies.^22,23^ Here, at a high Ca^2+^ of ∼20 mM, the distinct cohesion in cement cannot be explained by the DLVO theory, which predicts repulsive interactions between hydrated cement grains.^24,25^ Instead, at high salt concentrations, this strong attraction has been attributed to the ion-correlation mechanism.^9,13−17,19,24−28^ Recent works have shown that water immobilized near highly charged C–S–H surfaces reduces dielectric screening and enhances like-charge attraction (ionic correlation) at short surface separations.^25^

Although the interaction energy between C–S–H particles has been computed in many works, e.g., refs (25, 27−30), direct, distance-resolved force measurements of interactions between two C–S–H surfaces are scarce^19,20^ because of the challenges in preparing stable mineral surfaces in a symmetrical surface configuration. The available atomic force miscroscopy (AFM) measurements by Lesko et al.^19^ and Plassard et al.^20^ employ a C–S–H-covered silicon AFM tip against a C–S–H-covered flat substrate in aqueous inorganic salt solutions. Both works have reported that attractive forces act between two C–S–H surfaces in the presence of Ca-bearing solutions and under alkaline pH conditions. Purely repulsive interactions have only been detected at very low concentrations or in the absence of Ca^2+^ ions. These findings, along with the strong dependence of the measured adhesion on pH (and thus on C–S–H surface charge), point again to the dominant contribution of attractive Ca^2+^-associated ion-correlation forces, which arise due to like-charge electrostatic attraction.^25,26,29^ Although it has been established that dominantly attractive forces act between inert C–S–H at small separation distances of a few nanometers, it is also important to understand how the interaction forces evolve in the early stages of C_3_S hydration and C–S–H accumulation on its surfaces.

During C–S–H growth and densification, the chemical equilibrium in a highly confined interstitial pore cement solution changes, yielding a C–S–H nanoparticulate gel with highly heterogeneous microstructures.^31,32^ Although interlocking of ions and ion-bound water within nanometer-sized pores of the 3D C–S–H gel leads to net strongly attractive disjoining pressure between lamellar C–S–H nanoparticles,^24,33−35^ the magnitudes of attractive minima and repulsive steric maxima in the interparticle potential evolve in time, in correlation with the changing physicochemical conditions. Steric repulsion is important, especially during the initial stages of C–S–H growth, and contributes to more open and branched C–S–H microstructures.^36^ The properties of the cement paste immediately after mixing with water (i.e., workability, cohesiveness) depend on these interaction forces and evolve in time along with the densification of the hydrated product network (C–S–H and CH), until complete setting.^1,37^ However, experiments studying in situ C–S–H gel formation are still challenging and scarce.^35,38^

In this proof-of-concept study, we resolve early cohesion developing during in situ hydration of calcium silicate in rheological and surface force measurements. We first use small-amplitude oscillatory rheology (SAOS) to describe the structural buildup of reactive C_3_S and C–S–H pastes.^37,39−46^ This measurement is based on applying a (small) deformation below the elastic limit of the paste (“at rest”)^47^ and on following the evolution of cohesion (i.e., elastic modulus) with time. Thus, we can detect the development of interaction forces within the paste.^48^ We then demonstrate the preparation of thin films of C_3_S for nanoscale surface force apparatus (SFA) measurements. With SFA, we measure the forces acting between two opposing C_3_S surfaces upon their exposure to water. Our preliminary SFA measurements showed a direct correlation between in situ-captured surface growth and adhesion developing among water-exposed, dissolving, and reprecipitating C_3_S surfaces. The newly developed SFA setup with thin calcium silicate films grown by pulsed laser (PLD) supported by SAOS rheological measurements offers new perspectives to investigate the in situ C–S–H growth and surface forces simultaneously in a single-pore SFA geometry at different chemical conditions relevant to cement systems.

## Materials and Methods

### SAOS Rheological Measurements

#### C_3_S Paste

The tricalcium silicate paste was prepared by dispersing pure C_3_S powder (Sukgyung AT, Ca_3_SiO_5_, BET = 1.26 m^2^/g, mean size 3.15 μm; data provided by the manufacturer) with distilled water at ϕ = 44.6% (water-to-solid ratio w/s of 0.4). At this specific volume concentration, the paste shows an attractive gel-like behavior (i.e., elastic modulus *G*′ higher than the viscous one *G*″).

The C_3_S powder was slowly added (1 min) to the liquid while maintaining a low velocity speed (2800 rpm) in a vortex stirrer (Ultra Turrax TD300, IKA). Then, after scraping the tube walls, we mixed the suspension for 2 additional minutes, gradually reaching the stirrer maximum velocity of 6000 rpm. The resulting homogeneous paste is immediately charged on the rheometer geometry, and the test is started. The pH of the C_3_S suspension at w/s = 3 was measured through a pH meter (SevenCompact pH/cond S213 Mettler Toledo) with an electrode optimized for a highly alkaline solution. The calibration of the pH electrode was performed with three commercial buffer solutions in the pH range of 7.00–12.454. We estimate that, even if the absolute pH value may have an accuracy not better than 0.1, relative variations within a single measurement are significative down to 0.01. The electrode was inserted in the suspension immediately after mixing by hand the powder with water. The measure was conducted for 5 h, continuously stirring the solution with the use of a magnet. The pH evolves from 12.09 to 12.38 (after 3 h) due to the initial C_3_S dissolution, followed by a decrease due to portlandite (Ca(OH)_2_) precipitation, resulting in a final pH of 12.27 after 5 h.

#### C–S–H Gel

Calcium silicate hydrate C–S–H gel (composed of nanometric colloidal particles) was obtained following a procedure adapted from Myers and co-workers.^49^ MilliQ water was boiled in a flask to remove the CO_2_ and then cooled down for 10–15 min. Calcium oxide CaO was prepared by calcination of calcium hydroxide Ca(OH)_2_ (Merck) at 800 °C for 24 h. We subsequently mixed all ingredients—MilliQ water, colloidal silica SiO_2_ (Ludox TM50, Merck), and calcium oxide CaO, in this order—in a plastic container, with a water-to-solid mass ratio (w/s) of 45 and a Ca/Si = 1. The container was immediately sealed, placed on a roller (IKA Roller 6 basic, Werke GmbH and Co. KG) for a gentle stirring, and flushed with nitrogen for 20 min through two small holes made in the cover. The two holes were then sealed, and the container was kept on the roller to assure proper solution mixing and aging. After 1 week, the solution was centrifuged to obtain the gel. The final water-to-solid ratio was around 2, and this could slightly change due to the final solution centrifugation step. This procedure was validated by X-ray diffraction (XRD), which confirmed the presence of pure calcium silicate hydrate. The initial pH of the C–S–H suspension (at w/s = 3) was 12.2, which decreased slightly to 12.1 within 5 h due to carbonation.^50^

#### Rheological Protocol

Small-amplitude oscillatory rheological (SAOS) measurements were performed with a torque-controlled rheometer (MCR 302, Anton Paar). A specific plate–plate geometry was chosen for the two samples, taking into account their cohesion (i.e., elastic modulus, *G*′) and the tendency to create a slip layer on the upper plate (i.e., wall slip). In particular, the C_3_S paste was tested with a profiled plate–plate geometry (PP25/P2, top plate diameter 25 mm, Anton Paar), while the C–S–H gel was tested using with a sandblasted one (PP50S, top plate diameter 50 mm, Anton Paar). The C_3_S paste is in fact denser (i.e., it has a higher solid volume fraction) in comparison with the C–S–H gel and needs a smaller diameter dimension of the upper plate (i.e., 25 mm) to deal with higher normal forces developed by the paste in time while the cohesion increases. The use of serrated plates is required to prevent wall slip, a recurring problem with cement-based pastes. For the C–S–H gel instead, sandblasted surfaces are enough to assure contact (without any squeezing) and to avoid wall slip. For both samples, evaporation was prevented using a moisture-controlled chamber and by filling the groove on the bottom plate with tap water. The device temperature was set at 20 °C, and the gap between the plates was 2.7 mm for the C_3_S paste and 1.4 mm for the C–S–H gel. We performed two distinct oscillatory tests to define the elastic behavior of the samples and their reactivity in time.

The elastic regime is defined via an amplitude sweep (AS) measurement, in which a range of strain amplitudes (γ = 10^–3^–10^2^%) is imposed and the elastic (*G*′) and viscous (*G*″) moduli are measured.^47,51^ The end of elasticity (*G*_cr_^′^, γ_cr_) is defined as the point in which the value of *G*′ is reduced by 10–20% from its maximum values in the elastic plateau *G*_lin_^′^. For γ < γ_cr_, the range of strain deformation for which we observe a linear behavior is determined. The AS is preceded by a short time structuration (120 s) immediately after sample loading. Here, a small deformation in the range of linear elasticity for both systems (γ = 0.001% for the C_3_S paste and 0.005% for the C–S–H gel) was imposed. This preparation step was designed to avoid the initial noisy points related to the charging process and to the strong early reactivity of the C_3_S paste.

The second test consists of a long time structuration (TS)—analogous to the preparation step just described—to study the evolution of *G*′ (i.e., reactivity) of the two samples for ca 1.5 h.

All of the previously described oscillatory tests (AS, short, and long TS) were performed at a fixed frequency equal to 1 Hz, following typical SAOS measurements on cement pastes.^41,43^ Previous literature on these systems^39−41^ showed that the elastic modulus *G*′ is not affected by frequency variation. Frequency sweep tests (*f* = 0.01–100 Hz) at imposed low deformation (in the range of elasticity, γ ≤ 0.1%) were performed on the C–S–H gel; no variation was noticed in the values of both elastic and viscous moduli.

### Calcium Silicate Film Preparation

Thin films of calcium silicate needed for surface force measurements were grown by the pulsed laser deposition technique (PLD) using sintered tricalcium silicate pellets as PLD targets. At first, we prepared tricalcium silicate pellets by isostatic pressing of the C_3_S powder (Sukgyung AT) at 3000 bar, followed by pellet sintering at 1350 °C for 5 h in air. The established pellet preparation procedure did not alter the starting tricalcium silicate phase as verified by X-ray diffraction.

As substrates for PLD calcium silicate film deposition, we used ∼10 μm thick, freshly cleaved, high-purity, optical-grade mica sheets with a homogeneous deposition area of 1 × 1 cm^2^ (S&J Trading Inc.), prepared according to a standard surface force apparatus (SFA) mica preparation protocol.^52^ The films used for XRD characterization were deposited on thicker ∼50 μm mica substrates. The mica substrates intended for the SFA experiments were cleaved to a uniform thickness below 10 μm to yield transparent surfaces, which were subsequently back-coated with 35 nm of gold to allow interferometric measurements in the SFA. The calcium silicate films were deposited by PLD on the other side of the as-prepared mica substrates. PLD was carried out in a custom chamber equipped with a Lambda Compex Pro KrF excimer laser (248 nm) with 110 mJ pulse energy inside the PLD chamber and a spot size of 0.1 cm^2^. The PLD deposition temperature was 550 °C, and the background pressure was 0.04 mbar. We set a target–substrate distance of 5.5 cm and a deposition time of 30 min with a laser repetition rate of 5 Hz. The resultant film thickness was ∼300 nm.

### Calcium Silicate Film Characterization

The composition and surface properties of the PLD-deposited calcium silicate films have been analyzed by X-ray diffraction (XRD), X-ray photoelectron spectroscopy (XPS), scanning electron microscopy with energy-dispersive spectroscopy (SEM-EDS), and atomic force microscopy (AFM).

XRD characterization of the tricalcium silicate powder, the sintered target, and the PLD-deposited calcium silicate film on mica substrates was performed in the Bragg–Brentano configuration with a spinning sample stage on a PANalytical XPert PRO diffractometer, equipped with a Cu Kα X-ray tube (1.5406 Å). Grazing incidence XRD measurements of the calcium silicate films were carried out without sample rotation at an incidence angle of 2–4° on an Empyrean X-ray diffractometer (Malvern Panalytical) equipped with a parallel beam mirror on the incident beam (Cu Kα) side and a parallel-plate collimator on the diffracted beam side. All scans were done with a measuring time of 1 h per sample.

Near-surface chemical composition of the calcium silicate films was determined with XPS with an Axis Supra spectrometer from Kratos Analytical. We used an aluminum anode as the radiation source of Al Kα X-rays. As the inelastic mean free path (IMFP) of the ejected photoelectrons is only a few nanometers below the sample surface, we could obtain information about the chemical composition of the calcium silicate film without the influence of the underlying mica substrate. The exact IMFP depends on the material. In the course of the measurements, charge neutralization was used. The measured data was then evaluated using software CasaXPS, and the XPS photoemission peaks were fitted and quantified after subtracting a Tougaard-type background. The binding energy scale was calibrated by referencing on the C 1s peak, which was shifted to 284.8 eV.

SEM-EDS Ca mapping data were collected for the calcium silicate films using Hitachi SU5000 FE-SEM equipped with a Dual Bruker Quantax XFlash 30 EDS system. Before the measurement, the sample was coated with a 40 nm-thick layer of carbon with a Cressington 208C coater. The semiquantitative EDS spectra and element maps were collected at the applied voltage of 12 kV. High-resolution secondary electron (SE) imaging was performed with Tescan-Solaris FEG-SEM operating in the ultrahigh resolution (UHR) mode. Images were acquired using an accelerating voltage of 5 keV, a beam current of 300 pA, and a working distance of 5 mm.

AFM was performed with a Cypher ES atomic force microscope from Asylum Research. AFM topography images were collected in the tapping mode with ARROW-UHFAuD AFM probes supplied by NanoWorld. The calcium silicate surfaces were initially scanned in air. Subsequently, we injected about 1 mL of MilliQ water on top of the films so that both the sample and the AFM tip were submersed and followed the evolution of topography within the same region on a surface. The resultant images were processed in AR software by applying a 5 × 5 median filter. Roughness values were reported as root-mean-square (rms) values of the measured surface heights.

### Surface Force Apparatus Measurements

Surface force apparatus (SFA) measurements were performed using an in-house-modified SFA equipped with force measuring sensors. The use of strain gauge-type force sensors allowed us to obtain a real-time force signal that can be followed during the force measurements in addition to the standard multiple beam interferometry (MBI) measurements. A detailed design of the used SFA and the description of the force sensors have been provided in Wieser et al.^53^ Other relevant details of the SFA technique have been thoroughly described in Israelachvili et al.^54^ and Schwenzfeier et al.^55^

Here, calcium silicate films, PLD-deposited on gold back-coated mica substrates (Au-mica-calcium silicate), were glued to standard cylindrical SFA disks with a radius of curvature of 1 cm. For force measurements, two as-prepared calcium silicate surfaces were aligned in a cross-cylinder geometry with cylinder apexes rotated by 90° with respect to each other. All of our force measurements were performed using one set of calcium silicate surfaces in one 3 day-long SFA experiment. Forces were repeatedly measured by separating and bringing the two surfaces into contact.

We first measured forces between two calcium silicate films in air in a few contact positions to examine possible heterogeneity of the surfaces and to identify a contact area with relatively low surface roughness. Once a contact position with a relatively low roughness and no large particles visible in the optical SFA camera was chosen, we started measurements in water. We injected MilliQ water (the chamber volume was about 20 mL, and we rinsed the SFA liquid chamber 2 times with MilliQ water in the presence of surfaces before injecting the final 20 mL of water). Rinsing was performed to remove any potential loose particles from the liquid chamber while keeping the surfaces in close contact. In water, we measured forces for 3 days in the same contact region chosen in air as described above. Forces were measured as a function of distance in repeated approach–separation cycles. In each cycle, the maximum separation distance was set to ∼1 μm. During the SFA measurements, both surfaces were completely immersed in water to avoid any capillary effects. The solution was not replaced within this time, and solution evaporation from the SFA liquid cell was marginal. As such, we could monitor the measured surface forces in response to changes in film thickness, which were accessible based on the coupled interferometric measurements of the fringes of equal chromatic order (FECO). The concentration of ionic species at the end of the SFA measurements was determined with inductively coupled plasma mass spectrometry (ICP-MS; 7900 ICP-MS, Agilent Technologies, with argon carrier gas in the helium collision mode). We measured these concentrations in the solution sample extracted from the SFA chamber at the end of the 3 day-long experiment. The solution was filtered with a 0.2 μm filter, not diluted and not acidified. The final pH of the used MilliQ water was measured in bulk at the end of the 3 day-long SFA experiment, and it was 8.4. The starting pH of the MilliQ water was around 6–7, and we expect a local increase of pH in the solution confined between the surfaces during the SFA measurements (we used the Mettler Toledo InLab Micro pH electrode).

The SFA interferometric FECO data were analyzed using SFA explorer software.^55^ The thickness of mica substrates was calculated to be 3175.6 nm on both sides. The thickness of the PLD calcium silicate surfaces was calculated assuming the refractive index of calcium silicate of 1.72^56^ and was estimated to be ∼300 nm thick on each side. For further calculations, we assumed that the reprecipitating phase growing after contact with water has the same refractive index as that of calcium silicate, which provides sufficient accuracy in assessing the increase in layer thickness.

We report adhesion as the pull-off force, which corresponds to the maximum attractive force (force < 0) measured on retraction just before the adhesive jumps out of the surfaces from the contact (see the pull-off force marked in Figure 7B).

## Results and Discussion

### Rheology

**Figure 1:**
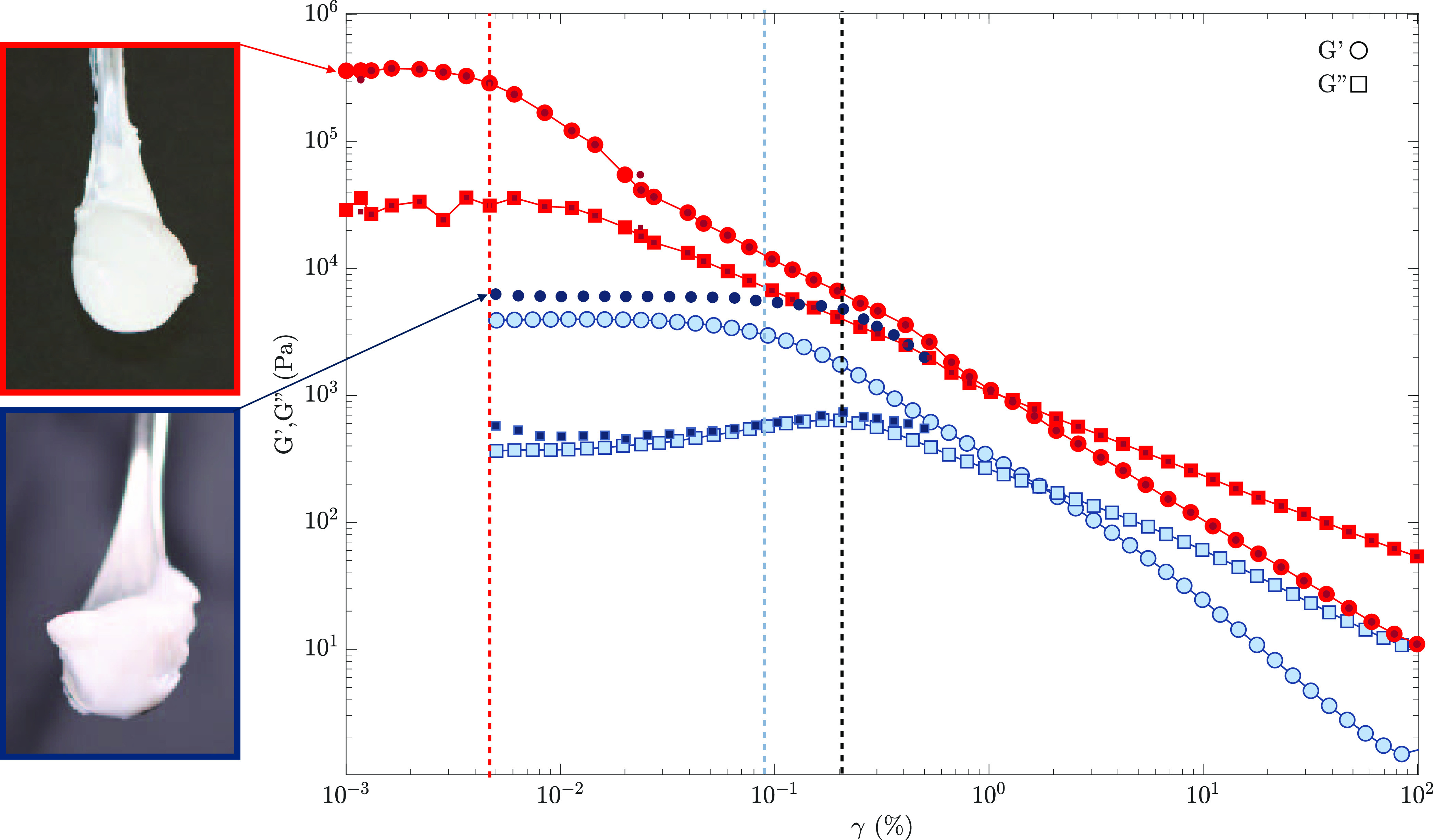
Amplitude sweep (AS) measurements on C_3_S pastes (dark and light red) and C–S–H gels (dark and light blue). The evolution of the elastic *G*′ (○) and viscous *G*″ (□) moduli is plotted as a function of the imposed amplitude strain γ (*f* = 1 Hz). The vertical lines indicate the elastic limit of the samples. The two pictures on the left correspond to the C_3_S paste (light-red frame) and the C–S–H gel (dark-blue frame) and were taken immediately after mixing.

In Figure 1, amplitude sweep measurements are shown on two samples of the C_3_S paste (two different mixes) and the C–S–H gel (two different centrifuged solutions). The vertical lines define the elastic domain (γ < γ_cr_) for all of the samples. The two pictures on the left, which were taken immediately after mixing (i.e., at the start of the experiment) from the same batches charged in the rheometer, show the consistency of the C_3_S paste and the C–S–H gel.

**Figure 2:**
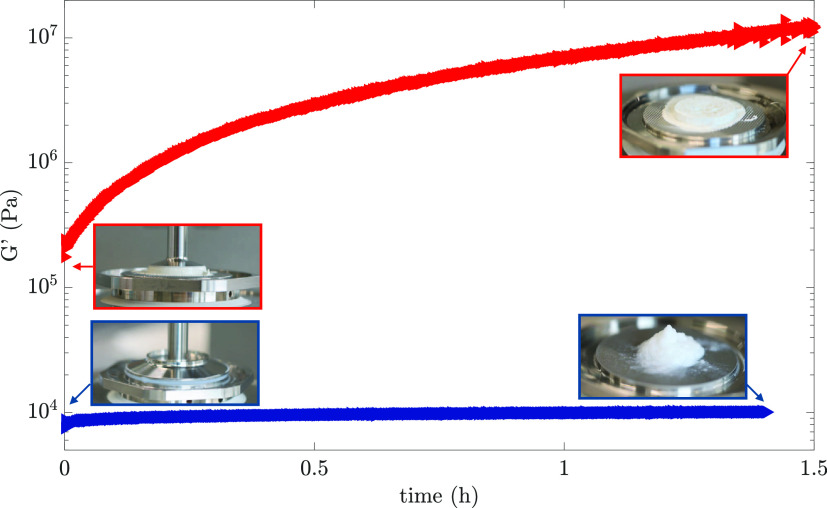
(Long) time structuration (TS) measurements on the C_3_S paste (red) and the C–S–H gel (blue). The evolution of the elastic modulus *G*′ is followed in time. The pictures correspond to the loading (left) and unloading (right) of the samples and have the same color code as the graphics.

The same color code is maintained for Figure 2, which shows the evolution of the *G*′ of the pure C_3_S paste and the +C–S–H gel in time (long TS, 5000 s, ca 1.5 h). Pictures of the plate–plate geometries were taken before and after the experiment during the loading and unloading of the samples. During all experiments, a moisture chamber was used to prevent evaporation.

Figure 1 shows a perfect overlapping of the two C_3_S samples (from two different mixes). In the case of C–S–H samples instead, the minor variation in the elastic domain is linked to the complex gel preparation and a slightly different solid-to-volume ratio due to solution centrifugation (resulting in a different gel cohesion, *G*′).

Both materials are viscoelastic, exhibiting an intermediate behavior between an ideal solid and a liquid with a transition after a specific deformation. At small strain amplitudes γ, *G*′ is higher than *G*″, revealing the solid-like behavior of both systems (“at rest”). After a certain value of the deformation amplitude (i.e., critical strain at the end of the elastic regime γ_cr_), both moduli drop and pastes end up in a liquid-like regime where *G*″ > *G*′. However, the values for which the elastic regime ends are different for the two samples. The C_3_S paste has a γ_cr_ ∼ 0.005%, almost 2 orders of magnitude lower than the one of C–S–H (γ_cr_ ∼ 0.1%). The difference in the extent of the elastic regime can be attributed to the different interactions and microstructures of the paste (i.e., more cohesive and fragile)^47^ and the gel (i.e., less cohesive and more elastic).^57,58^ Looking at the pictures on the left of Figure 1, we can notice that the macroscopic behavior of the two samples looked similar (both stick to the spatula without falling down). This is explained by their similar static yield stress τ_ys_ = *G*_cr_^′^·γ_cr_,^59^ around 10 Pa for the C_3_S paste and 8 Pa for the C–S–H gel. This means that, besides the difference in elasticity, we need to impose a similar stress on both samples to observe the transition from a solid- to a liquid-like behavior.

Next, by imposing a small deformation within this regime (γ = 0.001% for the C_3_S paste and γ = 0.005% for the C–S–H gel), the evolution of the elastic modulus (reactivity) is measured with time.

As we can see from Figure 2, the behavior of the two samples was completely different, as expected. In particular, for the C–S–H gel, we expected no structural variation (no reaction) at room temperature,^60^ resulting in a *G*′ almost constant in time. The C–S–H gel can undergo aging and drying once in contact with the atmosphere,^50^ but, as seen from the unloading picture, this was prevented by the moisture chamber. In general, C–S–H surfaces can be considered inert in the absence of ions.^22,23^ It was demonstrated that the C–S–H dilute suspension starts to show strong cohesion when the Ca^2+^ concentration is higher than 5 mM. The inert C–S–H paste shows a high elasticity due to the attractive forces (mainly arising from van der Waals interaction in the absence of salt ions^30^) acting between the nanoparticles and forming an interconnected 3D network.^28^

On the other hand, the *G*′ of the C_3_S paste increases continuously with time due to the dissolution–precipitation reaction upon contact with water.^51^ In the very beginning, the *G*′ of the paste is linked mainly with the high ion concentration in the pore solution due to the massive dissolution of C_3_S (initial low C–S–H precipitation), resulting in a very cohesive behavior at rest but fragile once sheared (γ ≥ γ_cr_). Following the hydration process, the continuous C–S–H particles’ precipitation starts to influence the overall contact area, which increases over time (contact aging^61^) until C–S–H percolation and final rigidification of the paste (setting). Thus, reprecipitated C–S–H acts as a nanocrystalline mineral glue, keeping the brittle calcium silicate grains together.

### Characterization of Calcium Silicate Films

**Figure 3:**
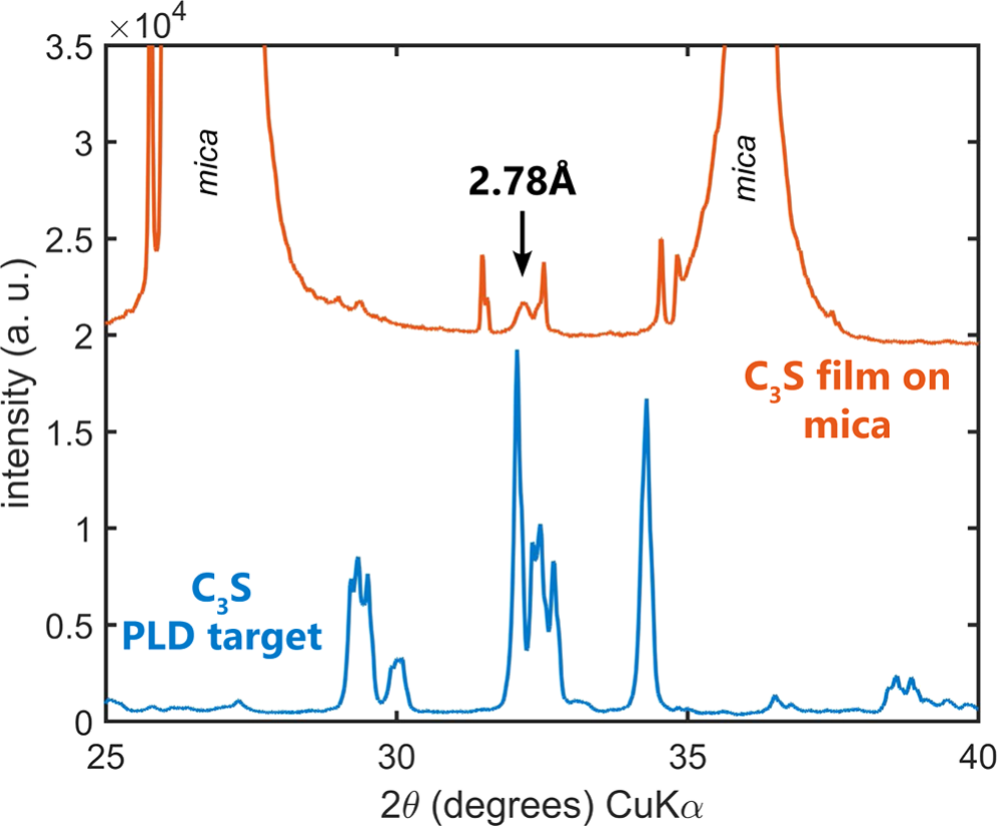
XRD diffraction patterns of the (bottom) C_3_S PLD sintered target and the (top) calcium silicate film PLD-deposited on a mica substrate. The sharp peaks in the film XRD correspond to mica substrate Bragg reflections stemming from Cu Kα,β and W-Lα_1_ wavelengths simultaneously.

The PLD-deposited thin films used in the later part of this study for SFA surface force measurements were thoroughly characterized to confirm the presence of a calcium silicate phase. Figure 3 shows the XRD patterns of the sintered PLD target and the resulting PLD-deposited thin calcium silicate films grown on the (001) lattice plane of mica. The PLD target’s XRD displayed peaks typical for a triclinic tricalcium silicate phase (C_3_S). The XRD pattern of the calcium silicate films was dominated by intense 00*l* reflections of the mica support, which are visible along with sharp spurious satellite peaks coming from reflections of the Cu Kβ radiation and of the W Kα radiation originating from anode contamination. Besides the sharp peaks related to mica, a broader peak at *d* = 2.78 Å was clearly detected (Figure 3). This peak position can be related to the most intense reflection of C_3_S (triclinic or monoclinic polymorph) or C_2_S (α-C_2_S, β-C_2_S). The broadening of the peak is consistent with a crystalline phase with small dimensions of the coherently diffracting domains and suggests the poorly crystalline nature of the deposited phase. We did not observe any diffraction peaks related to quartz (SiO_2_), lime (CaO), or portlandite (Ca(OH)_2_). It is worth noting that the intense peaks of the mica substrate dominate the measured pattern and peak overlapping with reflections of minor phases hampers phase identification. Nonetheless, on the basis of XRD data, it is confirmed that a calcium silicate crystalline phase is deposited on the mica support and that it is likely C_3_S. The XRD grazing incidence data show several peaks, which can be attributed to C_2_S and C_3_S at *d* = 3.32, 2.78, and 1.73 Å, in addition to a weak and broad reflection at *d* = 2.40 Å, which is compatible with C_2_S and C_3_S and also with CaO.

**Figure 4:**
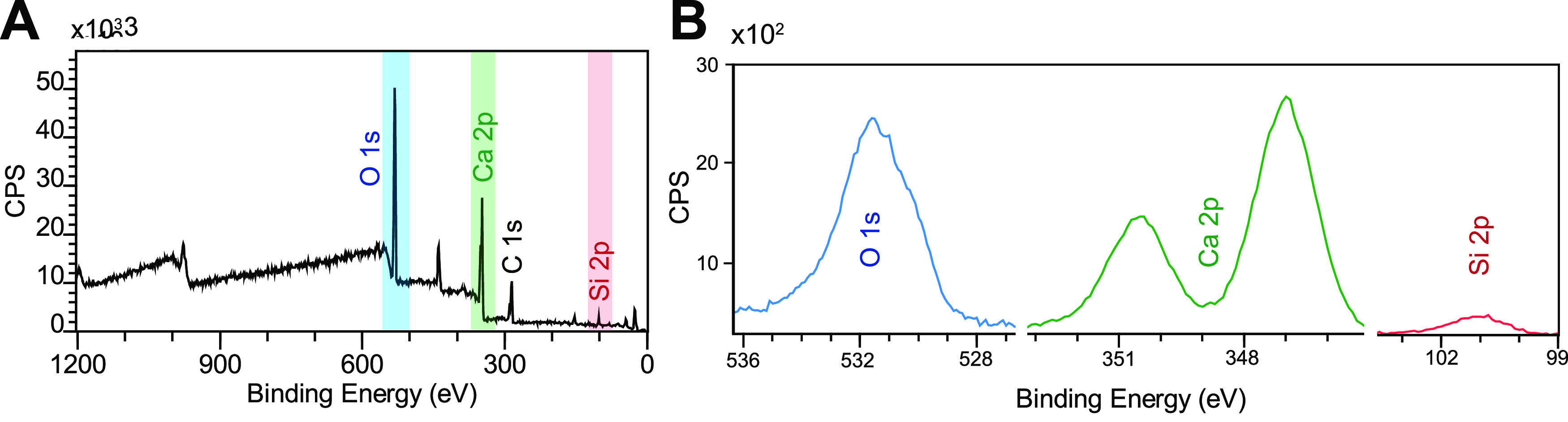
X-ray photoelectron spectroscopy (XPS) spectra of the PLD-deposited calcium silicate films. (A) Wide-scan XPS survey spectra with the main photoemission peaks used for the calcium silicate film composition semiquantification. (B) High-resolution photoemission peaks corresponding to O, Ca, and Si.

Surface-sensitive XPS measurements further confirm the presence of calcium silicate in the deposited PLD films. XPS survey spectra shown in Figure 4A reveal the presence of calcium, silicon, oxygen, and carbon photoemission peaks in the films. A semiquantification using the wide-scan survey spectra indicates that the ratio of Ca/Si was approximately equal to 2.5. The estimated element ratio was Ca_0.5_Si_0.2_O_1_C_0.8_, where C is adventitious organic carbon contamination. Since the depth of an XPS analysis is limited to several surface nanometers of the sample, we can exclude that the detected Si signal was related to the film-underlying mica substrate. This is additionally confirmed by the lack of K or Al signal, which would be present for the mica. High-resolution XPS photoemission peaks for O, Ca, and Si are shown in Figure 4B. The oxygen peak, centered at the binding energy (BE) of 531 eV, originates from the Si–O–Si structural units in the sample, whereas the asymmetric shoulder at a lower BE of ∼530 eV most likely corresponds to Si–O–Ca oxygen environments.^62^ The less evident asymmetry at ∼533.5 eV can be attributed to C–O or C=O bonds detected in the adventitious carbon layer. The Ca 2p photoemission peak showed a typical splitting associated with two spin–orbit components Ca 2p_1/3_ and Ca 2p_3/2_ and with maxima at 350.5 and 347.0 eV, respectively. In general, the BE of Ca shows little variation among common Ca-bearing compounds (such as CaO, CaCO_3_, Ca_3_(PO_4_)_2_) with a Ca 2p_3/2_ BE of ∼347 eV. This is also the case for C_2_S and C_3_S phases as reported by Black et al.^63^ The less intense Si signal was centered at the BE of 101.0 eV. A similar value of Si 2p BEs, ranging from 100 to 101 eV, has been reported for tricalcium and dicalcium silicate compounds.^63^ Si 2p photoemission originating from SiO_2_ or aluminosilicate minerals like mica usually displays significantly larger BEs of ∼103 eV,^64,65^ indicating that silicon oxide was not present in the deposited calcium silicate films and that the Si signal did not originate from the mica substrate.

**Figure 5:**
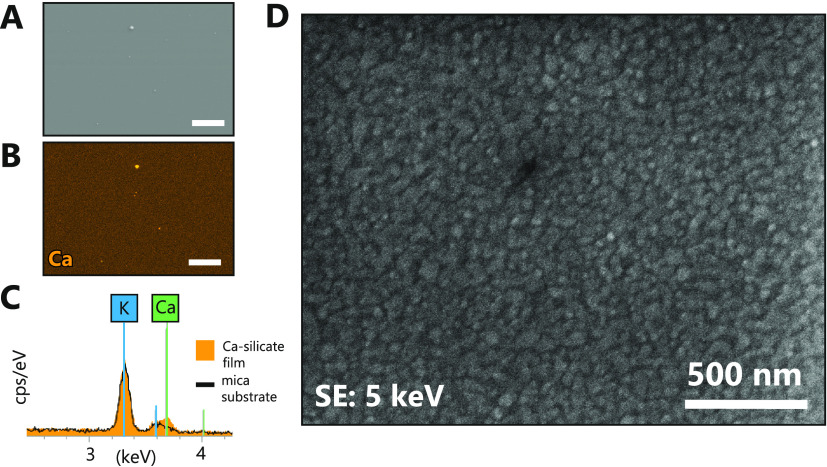
SEM-EDS semiquantitative Ca mapping on the ∼200 nm-thick calcium silicate film sample deposited on a mica substrate. (A) SEM SE image of the region chosen for the mapping, showing a flat topography with a few scattered μm-sized particles; the scale bar corresponds to 10 μm. (B) Ca EDS map of the region in panel (A) showing a largely uniform distribution of Ca as indicated by the orange color. A higher relative amount of Ca is measured for the μm-sized particles on the surface; the scale bar is 10 μm. (C) Fragment of a point EDS spectra showing Ca and K signals from a region on a calcium silicate film and on a bare mica substrate. (D) High-resolution secondary electron (SE) image of the calcium silicate film deposited on mica.

Distribution of Ca over micrometer-large film surface areas was analyzed with SEM-EDS, as shown in Figure 5A–C. We measured a very uniform distribution of Ca, suggesting that the deposited calcium silicate films are continuous over large areas. Ca was also abundant in larger micron-sized particles, occasionally detected on the surface. A more detailed high-magnification SEM SE image shown in Figure 5D revealed that the deposited calcium silicate material had a granular morphology and comprised ∼50 nm-large nanoparticles. We also noticed the presence of circular rims of larger micron-sized particles, locally contributing to a higher surface roughness.

**Figure 6:**
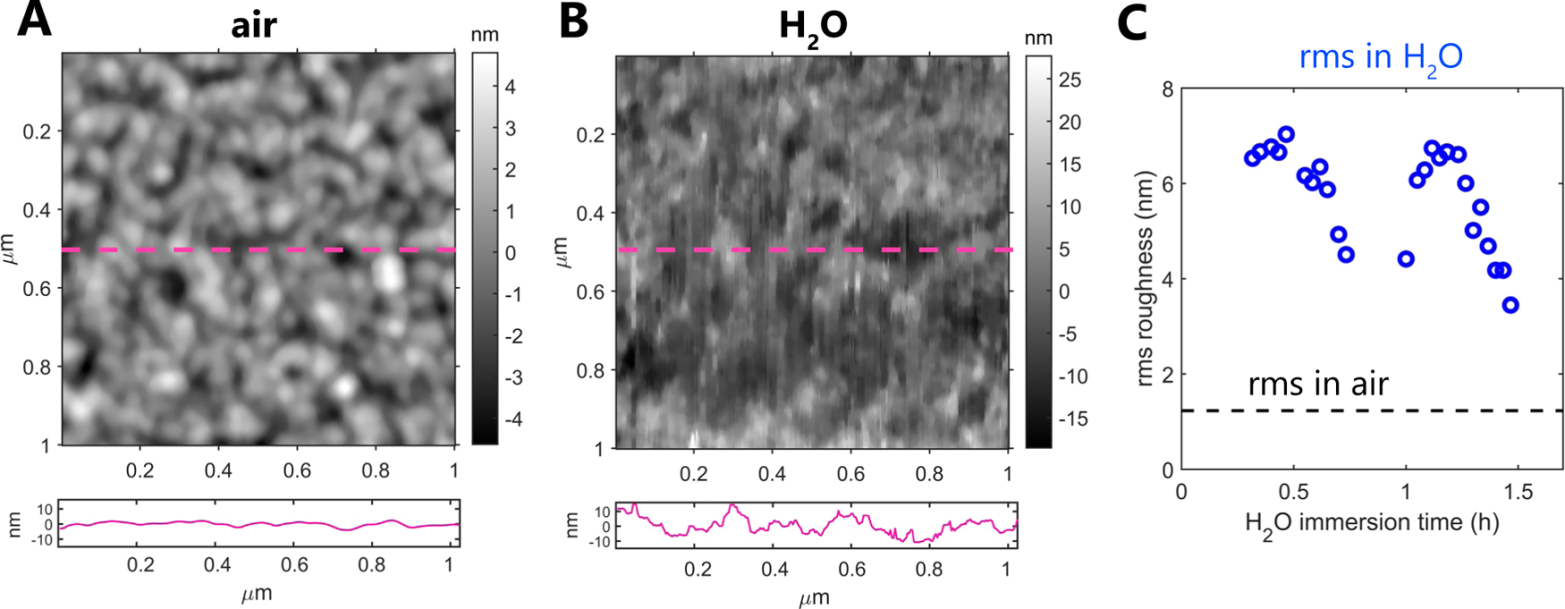
Atomic force microscopy topography maps of calcium silicate films in air (A) and in water ((B) sample immersed in H_2_O for 30 min). The panels below AFM maps show height profiles along the center of each AFM image as marked with a dashed magenta line. Note that the *y* axis is the same in both panels. (C) Comparison of the root-mean-square (rms) roughness measured in air and in water (over 1.5 h in the same position) for a 1 × 1 μm^2^ scan size. Each point corresponds to one AFM scan, including the measurement in air.

We further used AFM to study the nanoscale details of the film topography. The measurements performed in air revealed that the calcium silicate films are polycrystalline and are composed of uniform-sized nanograins, smaller than 100 nm in diameter (Figure 6A). At larger scan sizes, we also detected a significant amount of much larger, micron-sized particles that contribute to the quite high surface roughness; however, these were mostly located on sample edges, away from the PLD plume center. Subsequent AFM measurements in liquid confirmed that the films do not undergo full dissolution in water for several hours, as tested by continuously scanning the surface fully immersed in water as shown in Figure 6B. The rms roughness of the films in air was 1.2 nm (scan size 1 × 1 μm^2^), and it significantly increased upon exposure to H_2_O (rms up to 7 nm for a scan size of 1 × 1 μm^2^; see Figure 6C). We also detected a significant change in the film topography in water, with nanoparticles becoming less defined on a surface. This indicates that the films reprecipitated or swelled in contact with water, suggesting the gel-like character of the reprecipitated layer. However, despite the low thickness of the PLD-deposited films, there was no indication of complete dissolution–reprecipitation of the films: a smooth mica substrate topography that would indicate film dissolution was not exposed and a rough particle-laden surface was preserved throughout the whole measurement in water. In addition, there was no evidence of complete film dissolution in the SFA measurements; dissolution-related reduction in film thickness would have been indicated by the SFA-coupled white-light interferometric fringes. Therefore, the thin films behave as good model systems to study the early dissolution–reprecipitation phase by microscale surface force measurements.

### Surface Force Apparatus Force Measurements

**Figure 7:**
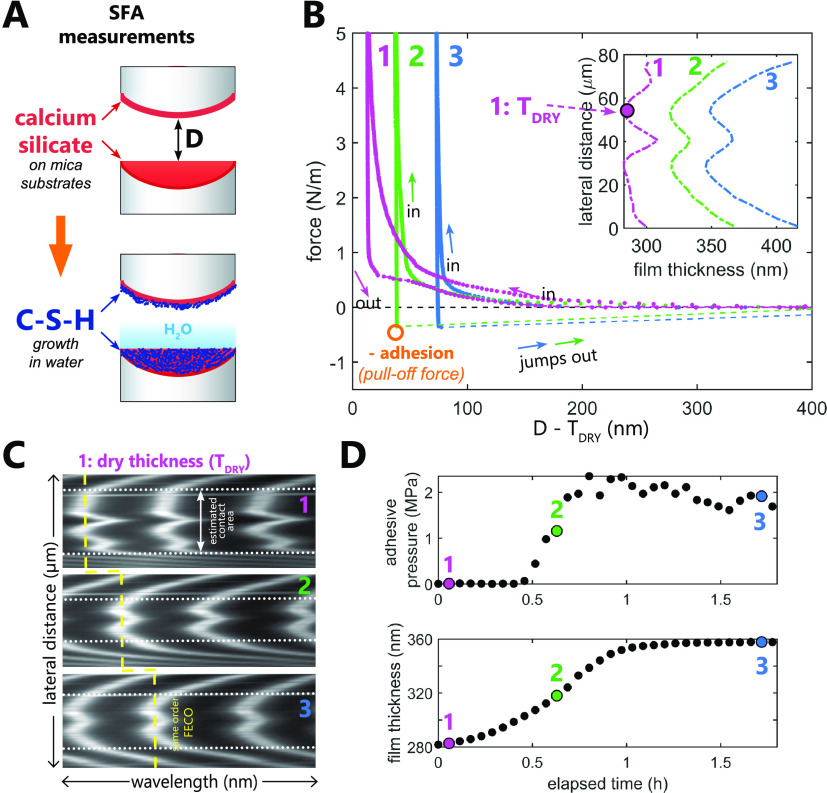
SFA measurements of forces between two opposing calcium silicate surfaces (C_3_S). (A) Schematic representation of SFA experiments, with C_3_S surfaces reprecipitating in water into calcium silicate hydrate (C–S–H). (B) Representative force–distance (*D*) SFA curves measured between reacting C_3_S surfaces. The hard wall position (i.e., separation distance at 0 nm) corresponds to the initial thickness of dry calcium silicate surfaces (*T*_DRY_). The separation distance is expressed as *D* – *T*_DRY_ to highlight the shift of the hard wall contact position with time that corresponds to the growth of C–S–H. The layer growth is correlated with the appearance of attractive force (force < 0) on separation. The inset shows the thickness increase and the change of the contact shape (extracted from the interferometric fringes shown in panel (C)). The approach and detachment rate was 100 nm/s for all force curves. (C) Shift of the interferometric FECO fringes at the hard wall contact position, corresponding to the growth of the C–S–H layer. The dashed yellow line marks the position of the FECO fringe of the same chromatic order. The white dotted line outlines the size of the contact region. (D) Adhesive pressure between calcium silicate surfaces and calcium silicate film thickness (on a single surface) as a function of elapsed time after the injection of water. Adhesive pressure is calculated as the pull-off force (see panel (B)) normalized by the nominal contact areas (see panel (C)). Further calculation details are included in the Materials and Methods section.

Having confirmed the presence of calcium silicate-like phases in the deposited PLD films, we subsequently investigated nanoscale surface forces acting between two opposing calcium silicate surfaces first in air and later upon their exposure to water, as sketched in Figure 7A. We performed all SFA measurements with the same pair of calcium silicate surfaces probed over 3 days. In our SFA measurements (Figure 7B) in air and for a few initial measurements in water, we detected only repulsive forces, likely owing to the high surface roughness.

The high initial surface roughness is evident from the topography-sensitive interferometric FECO fringes,^66^ as shown in Figure 7C. The irregular FECO fringes outline the shape of the contact area, indicating an initial variation of surface heights in air across the chosen contact region of ∼30 nm, as marked in the inset in Figure 7B. Although the rms roughness of the films as measured with AFM in air was only several nanometers, the FECO-derived roughness is comparable to the maximum peak-to-peak-valley height (the absolute value between the highest and lowest surface peaks) of ∼40 nm determined with AFM in water for a scan size of 1 × 1 μm^2^. In addition, in SFA, we have two rough films contacted across a large nominal contact area (with a diameter of ∼150 μm), with a high density of larger asperities on both sides.

The forces measured in air with the SFA were purely repulsive in several different locations on the surface (we moved the surfaces to probe several different contact regions until we picked one contact region with a relatively small roughness). However, during the subsequent measurements in water, we resolved water-induced and time-dependent adhesion (in this work, we define adhesion as a pull-off force: maximum negative force measured on retraction before surfaces jump out of the adhesive contact, as defined in Figure 7B) developing between two nanoparticulate calcium silicate surfaces. Despite the film roughness, adhesion started to become detectable ∼30 min after the water injection (see the more detailed evolution of measured forces in Figure S1). Note that it is possible that the small adhesion between the surfaces developed already earlier; however, it could have been below the detection limit of our measurements due to the high surface roughness of the PLD films. We followed the evolution of adhesive pull-off forces in water in the same contact region between the surfaces and observed that the increase of adhesion with time was correlated with the growth of the calcium silicate films (Figure 7D). This could be inferred from the significant shift in the hard wall contact position with time and thus the increasing thickness of the films (also evident from the change of the FECO wavelength position in contact; Figure 7C). Note that in Figure 7D adhesion is expressed as adhesive pressure (pull-off force divided by the contact area extracted from the flattened part of FECO fringes, as marked in panel (C)). We do that to account for the changing shape of the contact area within the 1st hour of the experiment. However, the real contact area is unknown due to the nanoscale roughness of the surfaces and the adhesive pressure is likely underestimated. In addition, FECO fringes and the view from the optical SFA camera both show no evidence of any significant material transfer from one surface to another or surface damage during the repeated pull-off events, indicating that the growing surface layers remain intact throughout the SFA experiment.

We interpret that this water-induced surface growth indicated by the SFA-coupled interferometric FECO measurements corresponds to hydration of C_3_S surfaces, with the nanocrystalline calcium silicate hydrate (C–S–H) gel and possibly crystalline portlandite, both comprising the main hydration reaction products.^67^ Precipitation is also evidenced in the optical SFA camera view as darkening of the surfaces, correlated with the surface growth indicated by FECO (Figure S2). Such uniform darkening, with no microscale features, points to the presence of a nanosized precipitate. We thus infer that water-reactive calcium silicate surfaces dissolve upon contact with water and reprecipitate immediately into common C_3_S hydration products, which are C–S–H and possibly portlandite. This reaction occurs locally, in the contact region between two C_3_S surfaces.

We performed SFA measurements in solid-to-liquid ratios much lower than in typical cement systems. However, our special SFA geometry comprises a narrow, wedge-shaped pore with a highly confined contact area over hundreds of micrometers. In such a geometry, it is possible to achieve supersaturation conditions with respect to C–S–H if precipitation is faster than diffusion of species out of the gap between two surfaces into the bulk water reservoir. Since we observe growth and reprecipitation of the surfaces, we infer that this is the case in our SFA experiment. A similar precipitation phenomenon has been demonstrated in our previous work, where amorphous calcium carbonate precipitates in the gap between two surfaces despite its undersaturation in the bulk solution.^68^ Here, the ionic concentrations measured at the end of our 3 day-long SFA experiment in the solution extracted from the SFA chamber reached 22 mM for Ca^2+^ and 12 mM for Si^4+^, which are within the range for C–S–H supersaturation conditions. However, they can be likely overestimated by possible dissolution of the films toward the end of the experiment and/or a partial loss of the reprecipitated C–S–H particles from the contact region, which at larger distances from the C_3_S may not be bound so strongly.^27^ The final pH of the solution used in the SFA was 8.4. This value is too low for typical C–S–H precipitation conditions; however, it is very likely lowered by carbonation of the solution due to CO_2_ dissolution from air. More data on ionic conditions and pH are needed in the future to better characterize the precipitation reaction. Although these are lacking, we access further properties of the reprecipitated hydration products in our subsequent SFA force measurements, as discussed below.

**Figure 8:**
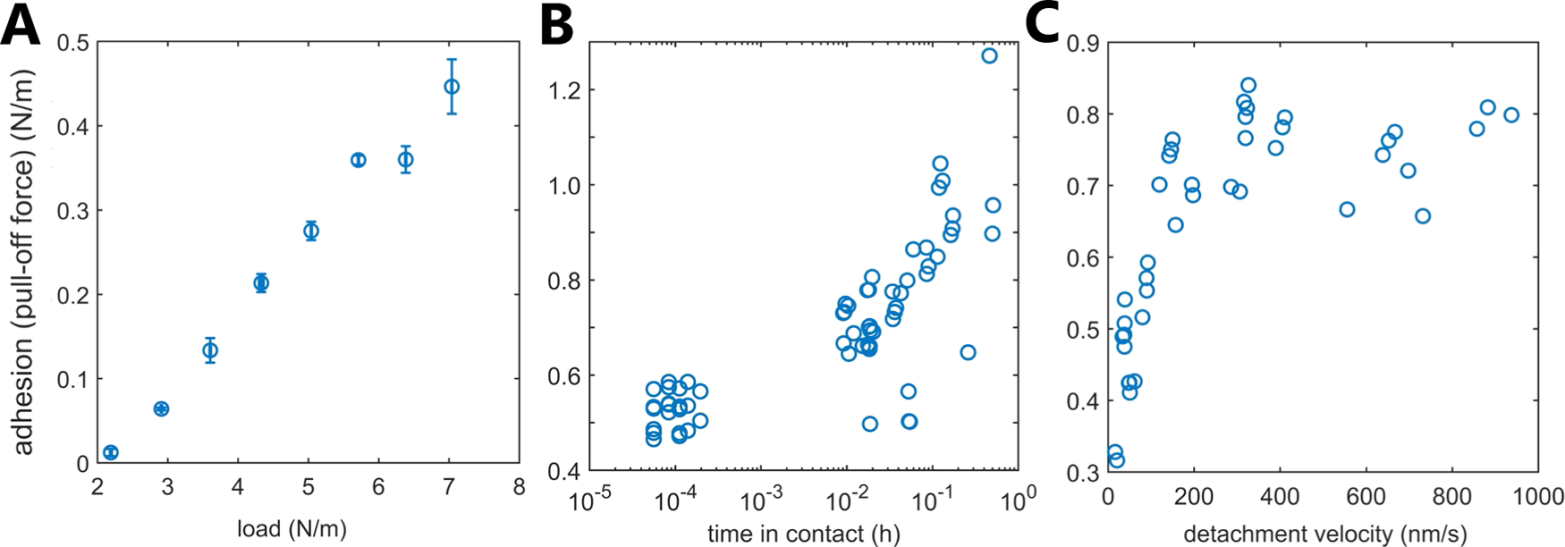
Adhesion (pull-off force) between two opposing calcium silicate surfaces measured in water in the surface force apparatus after a 3 h-long immersion in water. All data were collected in the same contact position (also the same as in Figure 1) after the layer growth ceased and surface thickness stabilized. (A) Adhesion as a function of applied load at constant approach and detachment velocity (100 nm/s), measured from high loads to low loads. Here, each data point corresponds to at least three loading–unloading cycles. (B) Adhesion as a function of time in contact at a constant load of 6000 mN/m and a constant approach and detachment velocity (100 nm/s). Dwell time was applied in a random order. Each data point corresponds to a single loading–unloading cycle. (C) Adhesion as a function of detachment velocity at a constant load of 6000 mN/m and no dwell time in contact. Approach velocity was varied in a random order. Each data point corresponds to a single loading–unloading cycle.

After 1 h in water (the forces were constantly measured in repeated approach–separation cycles in the same contact area in water), both the film thickness and adhesion stabilized (Figure 7D). Then, after 3 h, we investigated how the adhesion (pull-off force) depended on applied load, time in contact, and detachment velocity, as plotted in Figure 8. Although we never resolved any attractive forces on approach because of the high surface roughness of the films,^69^ this stable adhesion measured on retraction indicates robust attractive interactions between the reprecipitated surfaces once the surface asperities get deformed and are pressed into contact under a high applied load. Indeed, the measured adhesion was clearly load-dependent (Figure 8A). In addition, the adhesion increased when the surfaces were kept in contact at a constant applied load of 6 N/m for longer time durations (Figure 8B) and became stronger with the increasing detachment velocity at low velocity values (below 200 nm/s) but then stabilized at higher detachment velocities (see Figure 8C). The observed load dependence of adhesion can be in general related to the roughness of the surfaces: the surface asperities deform upon loading, leading to the increase of the real contact area between the surfaces. This is supported by the hysteresis between approach and retraction curves, where the energy is dissipated during deformation of surface asperities.^69^ Especially in our SFA force measurements with a soft, gel-like C–S–H material (Figures 7 and 8), load dependence is expected since a higher load produces more contacts between soft, compressible arrangements of nanoparticles on the two opposing surfaces. Thus, the higher roughness of soft, deformable reprecipitated C–S–H can lead to higher adhesion. The gel-like nature of the reprecipitated surfaces is further suggested by the fact that adhesion increased with increasing time in contact at a constant load (Figure 8B). Such dependence most likely means that the material is soft and mobile, can therefore deform, and is able to increase the number of contact points during the loading phase.^70,71^ Alternatively, this dwell-time-dependent adhesion can be related to chemical aging, i.e., formation of hydrogen bonds between silanol groups on the surfaces upon prolonged contact.^72^ Lastly, the adhesion was rate-dependent and increased with increasing detachment velocity (Figure 8C). This result suggests the viscoelastic nature of the soft, gel-like reprecipitated calcium silicate on our surfaces. Adhesion increase with the increasing rate of surface detachment has been commonly observed for viscoelastic polymer materials, and it is related to molecular-level rearrangements during contact healing and breaking,^73−75^ where the rate dependence is often caused by faster contact breaking at higher detachment velocities. Thus, at higher detachment rates, a larger fraction of energy is dissipated on the rearrangement of the material in the bulk, giving rise to a higher work of adhesion. The viscoelastic behavior has also been attributed to C–S–H phases in rheological measurements^60,76^ and confirmed by our SAOS measurements, as shown in the Results and Discussion section.

What is then the origin of the significant adhesion developing in SFA between gel-like, reprecipitated, hydrated calcium silicate surfaces in water? Based on our SFA measurements, we infer that the adhesion develops due to the change of the microstructure of the films, from brittle and rough C_3_S surfaces to soft, gel-forming reprecipitated C–S–H-like nanoparticles. Since force measurements detect the presence of a precipitate with viscoelastic properties, it is unlikely that there is a high proportion of brittle portlandite (CH, the main other C_3_S hydration product^67^) on the surfaces. The reprecipitated surfaces are laden with C–S–H nanoparticles (it has been shown that the density of C–S–H particles can be higher at the surface of reactive C_3_S grains^77^), which can create much more contact points when the surfaces are pressed together. This property of the C–S–H nanocrystalline gel can dramatically increase the real contact areas between the surfaces in the SFA and thus lead to higher measured pull-off adhesion. Importantly, this is only the case if the physicochemical conditions of the system allow for attraction between C–S–H particles and the C_3_S surfaces and between C–S–H particles themselves. Attractive forces have been shown to operate at distances lower than 3 nm at high pH, and thus, the high surface charges of both C_3_S and C–S–H are dominated by electrostatic ion correlation in the presence of divalent Ca^2+^.^25,27^

The fact that not only the C–S–H microstructure but also the high surface charges of C–S–H and Ca^2+^ are crucial for the robust cohesion of the system is highlighted by comparing it with a similar reprecipitation phenomenon of calcium carbonate. We have previously shown with the SFA that the viscous, amorphous calcium carbonate gel-like phase, which reprecipitated on two confined calcite surfaces, significantly increased the repulsion between these confining surfaces.^68^ In general, low-surface-charge calcium carbonate surfaces repel each other in water due to their strong surface hydration,^69,78,79^ and Ca^2+^ ions do not enhance attraction between calcite surfaces.^80^ In contrast, reprecipitation of C_3_S particles increases the local pH and produces highly charged C–S–H nanoplatelets, which attract each other mainly via ion-correlation forces in the presence of Ca^2+^^27^ (at pH above 11, most of the surface silanol groups are deprotonated^18^ and the surfaces undergo charge reversal due to Ca^2+^ adsorption). Here, in our SFA experiments, the C–S–H product remains on the surfaces despite repeated pull-off events and shows that the C–S–H layers are strongly linked to the unreacted C_3_S surfaces. The increase in the layer thickness of ∼80 nm on each surface (Figure 7D) suggests a multilayer accumulation of nanosized C–S–H (typically forming 5 nm-thick aggregates^81^) on reacting C_3_S grains. Assuming the preferred flat stacking of C–S–H (parallel to the C_3_S surfaces) at our solution conditions,^27,82^ this points to the high pH of the solution confined between the SFA surfaces. At low pH, the interaction between individual C–S–H particles likely becomes repulsive, as demonstrated by Delhorme et al.,^27^ and in our case would lead to progressive C–S–H removal from the contact region upon the repeated loading–unloading cycles. Such removal has been evidenced in our SFA geometry for the amorphous calcium carbonate gel, which had repulsive interactions with the confining mineral walls.^68^

Here, it is important to highlight that, as evidenced by SFA, C–S–H growth is not destructive. In many systems, mineral growth in pores rather causes stronger repulsion (most often due to developing crystallization pressure, which acts against the confining walls by pulling them apart^83^). Such volume-expanding mineral reactions exert stress on the confining walls, instead of sealing them together. This is, for example, very common for the hydration of Mg-bearing minerals.^84,85^

### Rheology and Surface Force Measurements

We studied the in situ hydration of calcium silicate in rheological and surface force measurements. Rheology provided macroscopic insights into the hydration reaction at a high solid-to-liquid ratio. SFA focused on the evolution of surface forces during the hydration reaction. In SFA, the reaction occurs locally in a highly confined pore, at low solid-to-liquid ratios. Both setups detected instantaneous reactivity of calcium silicate surfaces in contact with water, pointing to the C_3_S hydration and growth of C–S–H.

We showed that the rheological properties of the calcium silicate paste undergoing hydration reaction to yield C–S–H and a paste composed purely of the reaction products (only C–S–H) differ. The reactive paste was less elastic as the very initial cohesion is mainly linked to the high ion concentrations in the pore solution (massive C_3_S dissolution, low C–S–H precipitation). As the precipitation progressed, the contact (reactive) area increased and a C–S–H percolated network was created, keeping the brittle calcium silicate grains together, and providing additional cohesion to such a multicomponent system. The inert C–S–H paste instead was highly elastic, with individual particles forming attractive, porous, multiscale network-like structures, in line with another work.^28^ SFA measurements showed that interactions between two reactive calcium silicate surfaces became adhesive once there was a substantial amount of hydration product accumulated on the surfaces. Thus, weathered, hydrated C_3_S grains could be linked together by a soft nanoparticulate layer of C–S–H. Here in SFA, C–S–H layers adhered to the reactive C_3_S surfaces and the adhesion must have originated from the interaction of the C–S–H layers, as the thickness increase of ∼80 nm (Figure 7D) suggests multilayer C–S–H growth. Indeed, the adhesion reached its highest value at the maximum surface growth, which is in line with a previously reported higher cohesion at higher C–S–H volume fractions.^28^ In SFA, the contact between reacting C_3_S grains was repeatedly broken during the force measurements. Thus, SFA revealed that the forces between the hydration product-covered C_3_S remained adhesive over a long time during the hydration reaction, despite the progressive growth of the reprecipitating layer.

These findings in two differing systems confirm the robust early cohesive properties of C–S–H^19−21^ despite differences in the chemical composition of the interstitial/pore solutions in our two setups and despite possible variability in precipitated C–S–H crystallography, geometry, particle orientation, water content, and Ca/Si ratios.^10,27,28,81,86^ The existence of attractive interactions between precipitates at the early stage of calcium silicate hydration is the key to the percolation process in cement, where the final cohesion is provided by a connected network of intersecting hydration products.^87^ We suggest that the role of these attractive interactions is not only to provide cohesion in the system at all stages of hydration but also to limit the damaging crystallization pressure during the growth of C–S–H and especially brittle CH. The existence of net attractive forces between surfaces has been demonstrated to limit the crystallization pressure in the confined growth of calcium carbonate despite the much higher theoretical attainable pressure calculated from the thermodynamic limit.^88^

Although we prepared calcium silicate surfaces suitable for force measurements in a symmetrical surface configuration, we cannot directly interpret which types of interactions bring about the attraction between C–S–H-laden surfaces. More work under different chemical conditions is needed. Our SFA methodology with thin PLD-grown calcium silicate films can be used to further study surface forces between reactive calcium silicate surfaces. Force measurements with reactive surfaces are so far only possible to follow precisely with the surface force apparatus since this technique provides simultaneous information about surface layer growth and dissolution.

## Conclusions

In this proof-of-concept study, we used rheological and surface force apparatus measurements to follow the in situ hydration of tricalcium silicate surfaces. Small oscillatory rheological measurements (SAOSs) allowed us to follow the structuration (i.e., increase in cohesion) of the C_3_S paste in time and, as a result, gave us insights into the hydration process (C_3_S dissolution and C–S–H precipitation and contact). SAOS showed a strong cohesion developing between reactive C_3_S particles upon their transformation to C–S–H in contact with a high-pH aqueous solution and verified the viscoelastic and nonreactive nature of the C–S–H gel. Similarly, strong and lasting adhesion was developed between calcium silicate films in our precursory SFA measurements, which we interpreted to be due to the formation of a hydrated C–S–H-like phase upon contact with water. The adhesion was directly correlated with the increase in film thickness, pointing to the strong adhesive properties of the hydrating, reprecipitating layer. The rate-, load-, and time in contact-dependent adhesion pointed to the viscoelastic and gel-like character of the reprecipitated surface layer, in agreement with the C–S–H growth. The stable adhesion that was present despite the high surface roughness and increase in surface thickness confirmed the robust character of the C–S–H-induced cohesion. Although our measurements cannot directly distinguish the very nature of the interactions between C–S–H surfaces, we infer that in a large part it is related to the soft, gel-like microstructure of aggregated C–S–H particles, which enables large real contact areas (even for rough polyparticulate surfaces), allowing attractive forces, characteristic for the C–S–H system, to efficiently act between the reprecipitating surfaces.

## Supporting Information Available

Supporting force measurements; SFA camera images of surfaces before and after precipitation (PDF)

## Footnotes

a Cement chemistry notation C = CaO, S = SiO_2_, H = H_2_O
