# Supplementary material for: Detecting Early-Stage Cohesion Due to Calcium Silicate Hydration with Rheology and Surface Force Apparatus
Source: Langmuir. 2022 Nov 25;38(48):14988–5000. doi: 10.1021/acs.langmuir.2c02783 (PMC9730907; doi:10.1021/acs.langmuir.2c02783)
Supplement: Supplementary file 1 — la2c02783_si_001.pdf [file la2c02783_si_001.pdf]

Supporting Information for: Detecting early-stage cohesion due to calcium silicate hydration with rheology and surface forces apparatus

Teresa Liberto,[†] Andreas Nenning,[‡] Maurizio Bellotto,[¶] Maria Chiara Dalconi,[§] Dominik Dworschak,[‖] Lukas Kalchgruber,[‖] Agathe Robisson,[†] Markus Valtiner,[‖] and Joanna Dziadkowiec[*,‖,⊥]

†*Institute of Materials Technology, Building Physics and Construction Ecology, Faculty of Civil Engineering, Vienna University of Technology, Karlsplatz 13, 1040 Vienna, Austria*
‡*Institute of Chemical Technologies and Analytics, Vienna Institute of Technology, Getreidemarkt 9/E164, 1060 Wien, Austria*
¶*Opigeo SRL, Via dell'Industria 13, 36040 Grisignano di Zocco,Italy*
§*Department of Geoscience and Circe Center, University of Padua, via G. Gradenigo 6, Padova, Italy*
‖*Institute of Applied Physics, Vienna Institute of Technology, Wiedner Hauptstrasse 8-10/E134, 1040 Wien, Austria*
⊥*NJORD Centre, Department of Physics, University of Oslo, PO Box 1048, Oslo, Norway*

E-mail: joanna.dziadkowiec@mn.uio.no

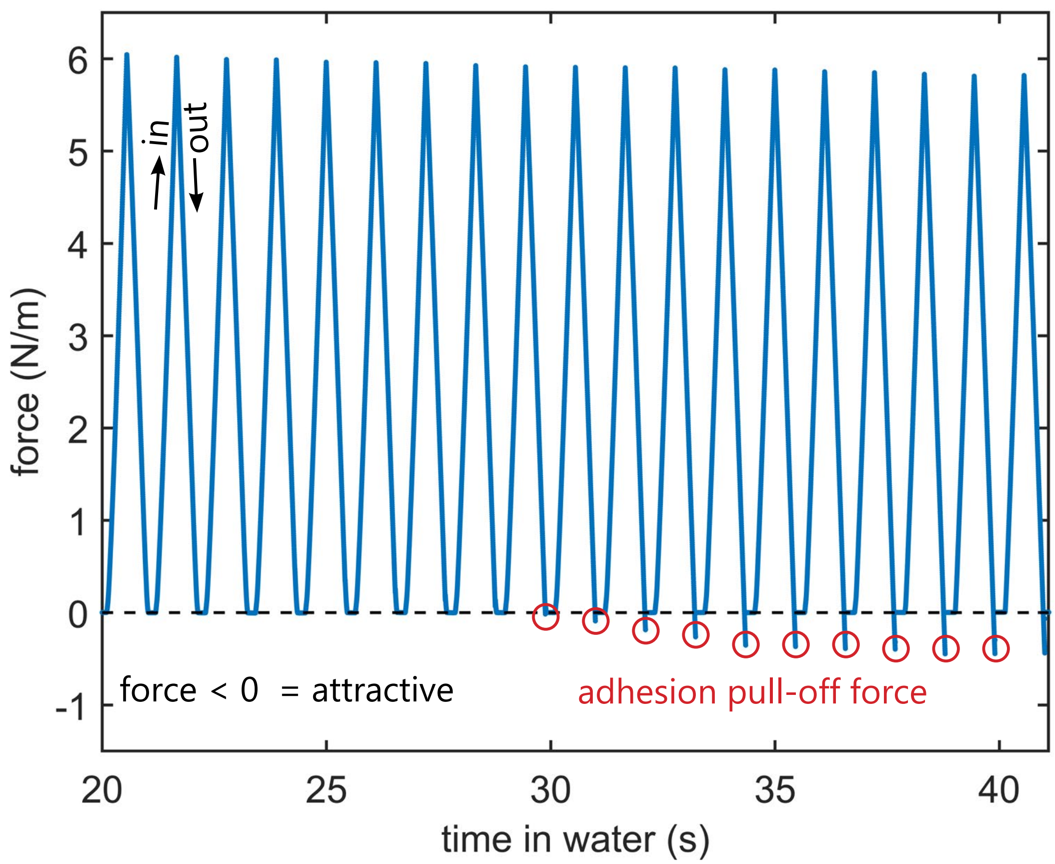

Figure S1: Forces measured with the SFA between two reactive calcium silicate surfaces after their immersion in MilliQ water. Forces are measured by constantly approaching and separating the surfaces. After 30 minutes of immersion in water, attractive forces on retraction (pull-off adhesion force) are detectable. Only strain gauge-based force signal is shown. Surfaces are separated by approximately 1 μm in each approach-retraction cycle.

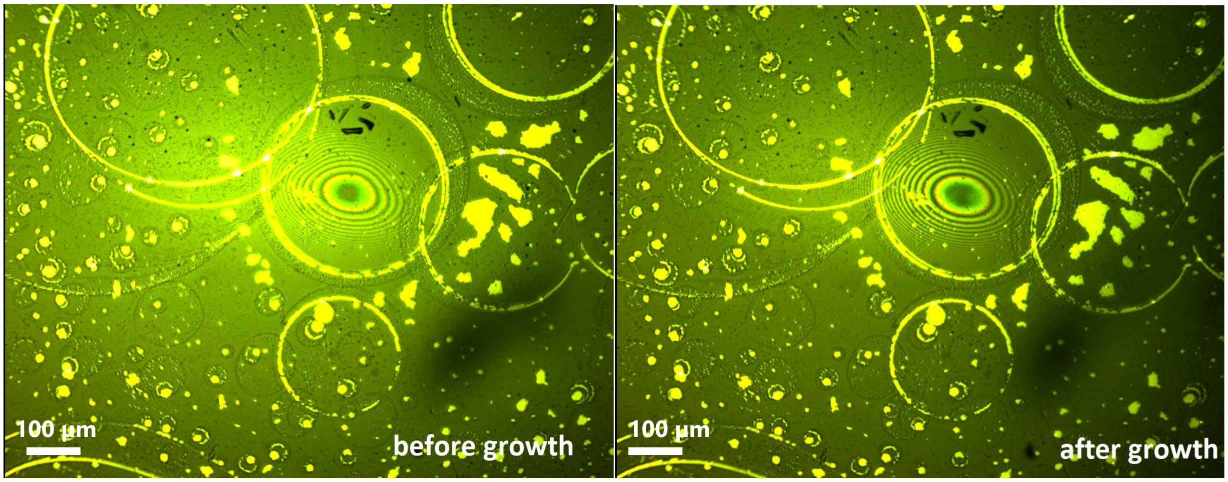

Figure S2: Reprecipitation of calcium silicate films upon exposure to water in the surface forces apparatus experiment (SFA). The images are from the SFA-attached top-view camera. The layer growth is evidenced as a slightly darker color of the whole imaged region. The surface darkening is correlated with the increase of the surface thickness as evidenced by the interferometric fringes of equal chromatic order. The contact region between two calcium silicate surfaces is outlined by Newton rings visible in the center. The other circular features are on the back side of the sample. These are defects in the gold layer mirrors enabling interferometric measurements underneath the mica supports (see Materials and Methods section for the details on SFA sample preparation).
